# Domestic chickens solving mirror-mediated spatial location tasks uncovering their cognitive abilities

**DOI:** 10.1038/s41598-024-64743-9

**Published:** 2024-06-19

**Authors:** Sonja Hillemacher, Inga Tiemann

**Affiliations:** Institute of Agricultural Engineering, University of Bonn, Nussallee 5, 53115 Bonn, Germany

**Keywords:** Animal behaviour, Zoology

## Abstract

The increasing demand on adapting modern livestock farming to higher animal welfare standards requires a thorough understanding of a species’ cognitive abilities to determine their adaptability. With the chicken being the world’s most numerous birds in animal production, it is mandatory to identify its cognitive abilities and limitations in order to meet its needs. We investigated if chickens are able to use and understand the reflective properties of a mirror that is the correlation of reflections of food rewards and their real location. In total, 18 female chickens of two different breeds were tested in a mirror-mediated spatial location task. Eleven out of eighteen hens solved the task successfully and thus, possibly exploited the correlation between the reflection in the mirror and the real food reward. We found differences on a breed and on the individual level, with different amounts of time needed learning the association of reward and mirror image. The results imply sophisticated cognitive abilities in chickens, assuming they may be able to understand how mirror images represent objects in the real environment, and to make use of it during foraging. The chicken’s cognitive ability might lead to a new understanding and provision of animal welfare-compliant production environments.

## Introduction

Mirror-image stimulation and mirror-image processing hold significant importance in the realm of comparative animal cognition, especially of spatial cognition, contributing to our comprehension of a species’ cognitive capacities. Since Gallup’s mark test^1^, a method that investigates if an animal is capable of mirror-self recognition (MSR) by drawing the correlation between its reflection in the mirror and its own body, MSR is thought to be the top end of mirror-image stimulation studies and a species’ level of cognition. But there are other testing procedures investigating on mirror-induced behaviours, which appear to be less complex than MSR, but allow for the classification of a species in a gradual model of mirror understanding^2,3^.

Among these testing procedures are e.g., mirror-triggered search or mirror-mediated object discrimination tasks. The former is a basic cognitive task in which the mirror is used as a cue that triggers search behaviour for desired items that are hidden in a fixed, familiar position and with their presence only visible in the mirror^4,5^. The latter investigates the animal’s capabilities of exploiting the correlation between an object, e.g. a food reward, and its reflection in the mirror^5,6^. This test method does not necessarily imply an animal understanding that the mirror image and the object are one and the same.

An enhanced and more sophisticated version of the mirror-mediated object discrimination task is the mirror-mediated spatial location task, in which the animal has to find a rewarding object hidden in a novel location and not, as in the mirror-mediated object discrimination task, in a familiar location^7^. Mirror-mediated spatial location tasks are a type of cognitive experiment designed to assess an animal’s ability to use mirrors as tools for spatial orientation and problem-solving. This test aims to investigate if an animal is capable of transferring knowledge about the relationship between an object’s reflection in the mirror and its exact location in the real world to a novel situation^7^. Up to now, solving mirror-mediated spatial location tasks and therefore, learning to associate an object’s reflection in a mirror and its spatial location in the real world has been reported for several different species including grey parrots^3,5^, several species of monkeys^4,6^, elephants^7^, as well as crows^8^.

For most of the time, research on animal cognition mainly focused on mammalian species^9^, while at the same time, avian species were declared as incapable of higher cognition due to their supposedly lacking cortex, for which the opposite has been demonstrated later^10,11^. In recent years, there is a growing number of studies proving the cognitive capabilities of different avian species to be underestimated^12^. Example, Eurasian magpies are discussed under caution to have passed the mirror mark test and thus being able of mirror-self recognition^13,14^, also Clark’s Nutcrackers are assumed to have passed a modified version of the mark test^15^, while Carrion crows^16^ and Hooded crows^17^ failed the mark test, adding to the controversial discussion on mirror-self recognition and the corresponding testing procedure. Although some species do not pass the mark test or do not show self-contingent behaviour in front of mirrors, these species might perform well in intermediate mirror-use tasks, such as in the mirror-mediated spatial location task^5,8^. Medina et al.^8^ showed, that although New Caledonian crows did not show self-directed behaviour in front of a mirror, they were able to pass a mirror-mediated spatial location task successfully.

New Caledonian crows are notorious for high cognitive performances, but an unlikely candidate, the domestic chicken, tends to be placed at the other end of the cognitive range, even though various studies have demonstrated surprising insights into their cognitive capabilities (see^18,19^, for detailed information). With nearly 26 billion animals worldwide in 2023^20^, the chicken (*Gallus gallus domesticus*) is the world’s most numerous birds and probably one of the most underestimated birds. Several studies on various aspects of their cognitive abilities challenge traditional stereotypes about these birds. Chickens have been shown to be capable of object permanence^21,22^, discriminating quantities and even having some basic form of ordinality and performing simple arithmetic operations^23,24^, estimating time intervals of at least 6 min comparable to toddlers^25^, possessing an episodic memory^26^, having some form of self-control^27^, transitive inference and logical reasoning^28^, complex referential communication in various social contexts^29,30^, and even having complex social cognition which is reflected in the ability of perspective taking^31^ and the use of social strategies^32,33^. Apart from the mentioned studies, there is one recent study demonstrating potential mirror-self recognition in chickens by using an ecologically embedded testing procedure^34^, indicating a sophisticated mirror-understanding in chickens.

In the current study, we investigated the ability of eighteen female chickens with two different genetic backgrounds (a native purebred breed, Japanese bantam (JB), and a commercial dual-purpose hybrid line, Lohmann Dual (LD)) to draw the correlation between a food reward’s reflection in the mirror and its position in the real world in a mirror-mediated spatial location task. The two breeds were selected due to their very different breeding histories: Lohmann Dual is a commercial dual-purpose hybrid line used for meat and egg production which underwent centuries of very intensive breeding history to reach certain performance parameters. On the other hand, the breed Japanese bantam is a native purebred breed and has been selected predominantly for tameness and a close animal-human relationship^35^, similar to game chickens. We were interested if there are differences in their cognitive abilities that might be a result of their different breeding background. We followed closely the experimental procedure of a study with New Caledonian crows^8^, but with a modified version of the test apparatus to better meet the chicken’s natural radius of motion and behaviour.

Considering the cognitive abilities of chickens that already have been revealed in other studies, we hypothesized that after explicit training chickens will be able to use the mirror as a tool to find a hidden food reward. Further, we hypothesized that there will be differences on a breed level and on the individual level with some individuals being able to use the mirror as a tool und some not, supporting the gradualist view on mirror understanding and mirror use^2^.

## Results

### Mirror-mediated spatial location task

For the whole sample of 18 hens, the mean number of successful trials (0.33 ± 0.08) was significantly higher than the level of chance (0.25; n = 18, t(17) = 4.306, *P* < 0.001), meaning they possibly used the mirror to locate the hidden food reward. Further, searches in the previously baited compartment was not a preferred strategy to find the food reward for the whole sample because it occurred significantly less frequently (0.21 ± 0.04) than it would be expected by chance (0.25; n = 18, t(17) =  − 5.261, *P* < 0.001).

Analysing breed effects, we find LD hens having on average significantly more successful trials (0.39 ± 0.1) than JB hens (0.30 ± 0.05; F(1,16) = 5.626, *P* = 0.031, partial η^2^ = 0.26), but breeds did not differ regarding the average number of searches in the previously baited compartment (LD: 0.20 ± 0.05, JB: 0.21 ± 0.03; F(1,16) = 0.290, *P* = 0.597, partial η^2^ = 0.018).

Also, breeds did not differ regarding the time they took to make their first step (latency: LD: 0.53 ± 0.42, JB: 1.03 ± 1.58; F(1,16) = 0.665, *P* = 0.427, partial η^2^ = 0.040) and the time they took until they retrieved the food reward (LB: 11.11 ± 4.16, JB: 15.69 ± 7.85; F(1,16) = 1.992, *P* = 0.177, η^2^ = 0.111).

**Figure 1:**
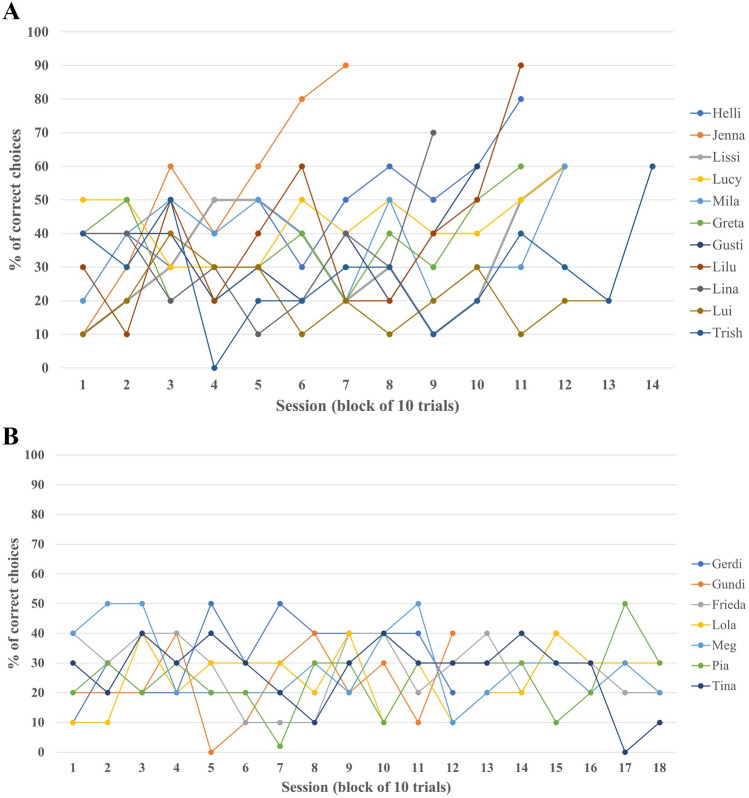
(**A**) Percentage of correct choices per session of each hen that reached the pre-established criterion. (**B**) Percentage of correct choices per session of each hen that did not reach the pre-established criterion.

The mirror-mediated spatial location task was solved successfully by 11 of 18 hens by using the food reward’s reflection in the mirror to locate the hidden food reward (see Fig. 1, Movie S1). A hen was considered using the mirror when it reached six or more successful trials within one session of ten trials (significantly above chance-level of 25%; see Table 1 for individual performances). The hens needed different numbers of sessions until they successfully used the mirror to locate the hidden food reward (see Fig. 1, Table 1). The individual hens used two main strategies to locate the hidden food bait: mirror-use and trial-and-error without mirror-use. The latter consisted of trial-and-error-strategy only, the strategy to search in the previously baited compartment corresponded to the level of chance (≤ 25%) for all hens.

**Table 1:** Performances of the individual hens during the mirror-mediated spatial location task.

Breed	ID	Sessions until meeting criterion	Successful trials in last session (meeting criterion)	Successful trials/session (M ± SD)	Searches in last baited compartment/sessions (M ± SD)	Time of latency (M + SD)	Time to food retrieval (M ± SD)
LD	Helli	8	6	4.13 ± 1.36	1.63 ± 1.06	0.59 ± 0.84	8.71 ± 6.76
LD	Jenna	3	6	3.33 ± 2.52	2.00 ± 1.73	0.83 ± 1.26	14.40 ± 8.14
LD	Lissi	12	6	3.25 ± 1.71	2.08 ± 1.38	1.36 ± 5.83	19.84 ± 36.21
LD	Lucy	12	6	4.33 ± 0.98	2.42 ± 0.90	0.65 ± 1.13	9.94 ± 7.05
LD	Mila	12	6	3.75 ± 1.36	2.08 ± 1.08	0.43 ± 1.07	10.40 ± 7.22
JB	Greta	11	6	3.73 ± 1.27	2.27 ± 1.35	0.05 ± 0.48	13.05 ± 15.21
JB	Gusti	10	6	3.50 ± 1.27	2.30 ± 0.95	0.55 ± 1.91	23.39 ± 35.87
JB	Lilu	6	6	3.50 ± 1.87	2.33 ± 1.21	1.15 ± 2.51	26.31 ± 30.29
JB	Lina	9	7	3.33 ± 1.73	1.33 ± 0.87	0.07 ± 0.63	11.17 ± 10.87
JB	Lui	14	6	2.36 ± 1.39	2.07 ± 0.83	0.00 ± 0.00	8.44 ± 8.21
JB	Trish	14	6	2.86 ± 1.56	2.29 ± 1.14	0.60 ± 3.98	8.96 ± 9.61
LD	Gerdi	(12)	(2)	3.25 ± 1.29	2.17 ± 1.53	0.22 ± 0.52	7.38 ± 5.86
LD	Gundi	(12)	(4)	2.33 ± 1.30	2.50 ± 1.38	0.06 ± 0.40	12.00 ± 19.23
JB	Frieda	(18)	(2)	2.78 ± 1.04	2.17 ± 0.94	0.83 ± 7.34	12.26 ± 15.99
JB	Lola	(18)	(3)	2.43 ± 0.94	2.39 ± 0.94	0.35 ± 0.66	10.23 ± 10.94
JB	Meg	(18)	(2)	2.89 ± 1.20	2.00 ± 0.88	0.20 ± 0.62	35.05 ± 50.51
JB	Pia	(18)	(3)	2.50 ± 0.89	2.40 ± 0.82	2.58 ± 2.99	14.11 ± 18.96
JB	Tina	(18)	(1)	2.70 ± 1.15	2.22 ± 1.04	5.28 ± 19.95	16.45 ± 23.97

Presented are the individual data of all hens of the two breeds (Japanese bantam (JB) and Lohmann Dual (LD)) including the required numbers of sessions until the individual hens reached the criterion, numbers of successful trials when hens met the criterion to solve the task successfully, mean numbers of successful trials per session and mean searches in the last baited compartment per session, times until food retrieval and latencies until first step. Bracketed italic numbers indicate hens of the no-mirror-use group that did not reach the criterion.

Hens that solved the task successfully needed between 3 (Jenna) and 14 sessions (Lui and Trish) until they successfully learned to use the mirror to locate the hidden food reward. Mean numbers of successful trials per session ranged between 2.33 ± 1.30 successful trials/session (Gundi) and 4.33 ± 0.98 successful trials/session (Lucy). Some hens started walking towards the test apparatus the exact moment they were placed inside the arena (e.g., Lui with 0.00 s ± 0.00 s), while others took quite long until they made their first step (Tina: 5.28 s ± 19.95 s). Interestingly, the hen that was on average the fastest in finding and extracting the food reward (7.38 s ± 5.86 s), was one that did not solve the task successfully and relied on trial-and-error-strategy only, but with a high speed. Others took quite a while until they found and extracted the food reward (e.g., Meg with 35.05 s ± 50.51 s).

### Control with non-reflective surface

In this session, the mirror was reversed in a way that the animals could only see the non-reflective surface. With the two-box-apparatus it was tested if they use other cues than the mirror to locate the hidden food reward. The probability to find the food reward by chance with first try was at 50% in the control session with the non-reflective surface. The number of successful control trials (first choice = correct choice) corresponded to the level of chance (2-sided binomial test: *P* = 0.311). Ten of the eighteen hens (4 LD and 6 JB) found the baited compartment with their first try in the first control trial. In the second control trial, eleven of eighteen hens (4 LD and 7 JB) found the baited compartment with first try.

## Discussion

The mirror-mediated spatial location task investigates sophisticated cognitive abilities in animals, even though its complexity is classified below mirror-self-recognition tasks^5^. And although the proportion of studies on the cognitive abilities of birds, especially of corvidae and parrot species, has skyrocketed in recent years^12,36^, the world’s most numerous bird species, the chicken, has mostly been out of focus when asking the big questions in cognitive science—do chickens possess skills in mirror-image processing or even some level of self-awareness? The results of our study indicate that chickens can solve mirror-mediated spatial location tasks successfully after explicit training, but not every chicken is able to make use of the reflective properties of a mirror. The performance differs on an individual level and results in most chickens passing the test. Eleven of the eighteen chickens tested were able to use a vertical mirror to locate a hidden food reward that was only visible in the mirror and thus, successfully associated the two-dimensional mirror image with the location of the three-dimensional object in the real world.

Our results suggest that the chickens successfully employed some form of associative learning. However, they do not demonstrate whether chickens possess the ability to actually comprehend that the reflected mirror image and the corresponding object in the real world are one and the same, although we cannot rule out this possibility. Studies by Medina et al.^8^ and Hill et al.^40^ also noted in their research on crows and on sea lions that their similar results may be originated from associative learning. Instead of using the mirror image functionally and thus, understanding the correlation between the two-dimensional representation and the three-dimensional object in the real word, it may also be possible that the sea lions and crows, as well as our chickens, formed a conditioned association or rule to detect the reward.

On a population level, we conclude chickens are able to successfully locate the hidden food reward in one of the four boxes of the spatial location task by using the food reward’s reflection in the mirror after explicit training. Use of the mirror was in most cases visually clearly observable; the hen elongated her neck to have a better view on the mirror-image and moved along the four-box apparatus constantly checking the spatial contingency between her position in front of the compartments’ closed hatches and the reflection of the food reward in the mirror (see Movie S1).

We found differences on the breed level, indicating that LD hens had on average more successful trials than JB hens, but these results have to be interpreted with caution as the sample size was different for both breeds (7 LD hens and 11 JB hens) and in general rather sample (18 hens in total). It would be interesting to further explore the cognitive abilities of chickens, considering their diverse genetic backgrounds and breeding histories. Understanding any potential differences attributed to their distinct breeding purposes could provide valuable insights into their cognitive capacities.

On the individual level, not all chickens passed the mirror-mediated spatial location task successfully and those who did, needed different amounts of time (numbers of sessions). These findings contribute to the research focus of personality and capabilities at the individual level within a species’ behaviour in mirror-image processing tests in detail, as well as animal behaviour studies in general^1,13,34,37–39^.

Ranking the hens’ performance in the mirror-mediated spatial location task within the existing literature on other species that either solved or did not solve similar tasks is challenging. This difficulty arises because most studies on similar tasks employed markedly different experimental procedures, including varying amounts of prior training, different numbers of trials per session, and distinct total numbers of trials before concluding the experiment^4,5,8,37,40,41^. While, e.g., New Caledonian crows^8^ needed between 2 and 3 sessions until they were able to use the mirror to locate the hidden food, sea lions learned to use the mirror after 4 to 5 sessions but following a different experimental procedure^40^. Hens of our study needed between 3 and 14 sessions, with an average of 10 sessions, until they started to use the reflective properties of the mirror. While this is an inferior performance than e.g., the crows’ one, it does not detract from the result that chickens are able to solve the task successfully. The performance of the hens might have been better when each trial would have been stopped immediately after their first decision, regardless of if it being the correct decision or not. This is what Pepperberg et al.^5^ did in their experiments. Thus, the motivation of the animals to use the mirror might be higher when they only get the food reward when they actually use the mirror. In our case, the hens got the food reward in each trial, either by using the mirror or by using simply trial-and-error strategy, which might have decreased their motivation to use the mirror. The hens’ performance may indicate that chickens should be ranked behind New Caledonian crows regarding their cognitive abilities with mirrors—or/and their comprehension. To our knowledge, in all studies on responses to mirrors, the animals need various amounts of prior mirror-exposure to identify the reflective properties of the mirror, which is probably because in their natural environment, most animals are hardly exposed to mirrors or their own mirror image. Training and learning seem to be essential parts of mirror-image processing tasks.

When the mirror was introduced for the first time, during the 5th habituation session, some of the hens (n = 9) responded with aggressive postures or attacks to their mirror-image or tried to reach the food reward through the mirror by pecking at the reflection in the mirror (n = 5). The display of social responses towards the own mirror image is part of mirror habituation in most other species^5,8,40,42–44^. The transition of social responses to more self-directed responses during extended mirror exposure is seen as the prerequisite for MSR^45^.

Ünver et al.^46^ criticized that most studies on using mirrors to locate hidden food do not account for the peripheral field of vision of the subject in question, as many studies aim to give as little restriction to the animal as possible when responding to the mirror image. This remark has been considered here by designing our experimental apparatus in a way that prevented the animals from seeing the food reward in any other way than by its reflection in the mirror or by inserting the head through the corresponding hatch. Other controversy addresses the interpretation of the outcome of these testing methods, because results might simply rely on mirror-triggered searching behaviour rather than on animals using the mirror for information about where to find the food^5^. Here, the behaviour of the chickens during the control trial with the non-reflective surface proved vividly that the searching behaviour of the chickens was not triggered by the mirror but was rather based on an innate high exploratory behaviour and motivation to find the food reward.

Our results are in line with a recent study indicating MSR in roosters^34^ and add to the necessity of performing multiple mirror tasks with varying parameters to bring the multidimensional phenomenon of self-awareness to light^12,47^. Most notably, the results imply that the chickens’ level of cognition might be closer to that of crows and other ‘conscious’ bird species than originally thought.

Our results provide compelling evidence for the chicken’s enhanced cognitive skills, which are an essential puzzle piece to the question of its mental needs. Unmet needs, mental and physical, may affect and alter the mental state of an animal negatively and result in an indisposition^48^, and thus a reduced level of welfare^49^. For the management of poultry, as well as for the design of the husbandry environment, we have to be aware of the cognitive abilities and resulting mental needs of the animals. The growing amount of evidence on the various cognitive capabilities of chickens leads to a change in the ethical approach and handling of this species. Understanding an animal’s ability to engage with mirror-mediated tasks has implications for animal welfare and enrichment, leading e.g., to cognitive enrichment and other opportunities for mental stimulation.

However, some methodological limitations of the current study have to be discussed. On the one hand, the mirror-mediated spatial location task is mainly characterized by the novelty of the location where the desired object is hidden or by the novelty of the desired object itself^7^. Therefore, it remains unclear if repeated testing may result in habituation to the test apparatus and thus, makes the transfer measurable. Therefore, future work should focus more intensively on a prolonged training period to preserve novelty of the actual test design. On the other hand, the test outcome does only account for the animals being able to associate the reflection in the mirror with the reward in the real world, but it does not prove that the animals did actually develop a deeper understanding of mirror correspondence that reflection and reward are one and the same object. Thus, some kind of control experiments with a different stimulus than the mirror, e.g., a light turning on in correspondence with the rewarded compartment to disentangle conditioned learning from specific image processing. Additionally, the reward could be changed in the final spatial location task to boost the challenge^40^.

Among the species that did not pass a mirror self-recognition test (mark test), but did perform well in mirror-mediated object discrimination tasks or mirror-mediated spatial location tasks are several monkey species^4,50,51^, gorillas^52^, pigs^37,38^, sea lions^40^, African Grey parrots^5^ and, as previously mentioned, New Caledonian crows^8^. These studies, as well as our results, add to the controversial discussion on interpreting mirror self-recognition and mirror-image processing skills as the so far binary model rather than a gradualist model. The gradualist model concedes different species different levels of self-awareness ranging from low to intermediate to high levels of mirror understanding as proposed by de Waal^2^. With our study, we want to address and extend the gradualist model on self-awareness and mirror self-recognition proposed by de Waal^2^ to mirror-image processing skills in general, with two underlying dimensions: an individual and a population-level. The individual animal might learn to exploit information of mirror-images through experience with the mirror, while among a population, there might be individuals who show more or less sophisticated mirror-image processing skills^53,54^, due to more or less experiences or a neuronal surplus enabling the animal for a faster learning progress and flexible cognitive abilities^55^.

It's important to note that intelligence can manifest in various ways, and measurements may differ across species. Chickens, despite their reputation as “dumb” farm animals, display a range of cognitive abilities that continue to be a subject of scientific investigation. Researchers are continually uncovering more evidence of their cognitive capacities and challenging preconceived notions about the cognitive capacities of poultry. The implementation and complementation of such knowledge in the assessment of animal welfare^56,57^, the management of these animals and the design of husbandry and production systems, would enhance animal welfare standards to a new level.

## Methods

### Tests subjects

Eighteen adult (2 years old), female chickens with two different genetic backgrounds (n = 18, 11 hens of the purebred breed “Japanese Bantam” (JB) and 7 hens of the commercial dual-purpose hybrid “Lohmann Dual” (LD)) were included in this study. All animals hatched and were reared and kept in mixed-gender groups on-farm at the Poultry Research Centre, Rhein–Kreis–Neuss, Germany. Hens were individually marked with coloured leg bands and were naïve to any experimental procedure prior to the experiment. Housing conditions met the requirements of a conventional free-range housing system, with animals having access to a barn (6 m^2^), provided with feeder, water, nests and perches, and an outdoor area (156 m^2^). Animals were fed for ad libitum intake (all-mash VoMiGo, Deuka, Deutsche Tiernahrung Cremer, Düsseldorf, Germany) including water and grit. The animals were under supervision of the veterinarian in charge and have been vaccinated against coccidiosis, infectious bronchitis and Newcastle disease (the last two repeated every 3 months).

### Ethics declaration

The keeping of the animals complied with the order on the protection of animals and the keeping of production animals in Germany^58^. Animals were raised from hatch on and kept at the Poultry Research Centre, Rhein–Kreis–Neuss, Germany, throughout the duration of the experiment. The study was approved by the local animal welfare committee of the University of Bonn. All methods were carried out in accordance with relevant guidelines and regulations. After the experiment, hens remained on farm for breeding purposes or were given to private breeders supporting animal genetic resources. The authors comply with the ARRIVE guidelines^59^.

### Experimental design

The whole experiment took place in an arena with the dimensions 180 × 180 × 72 cm (L × D × H) in a separate room, which was evenly illuminated by a daylight emitting fluorescent tube with UV-array and electronic ballast unit to increase flicker frequency. The arena was built of grey Trovidur®, the floor was covered with a panel with green lacquer foil made of water-resistant vinyl on top. A camera was attached to the ceiling, centrally above the arena, and connected to a computer. All sessions were recorded on video using the software Viewer 3.0.1.241 (Biobserve GmbH, Bonn Germany).

**Figure 2:**
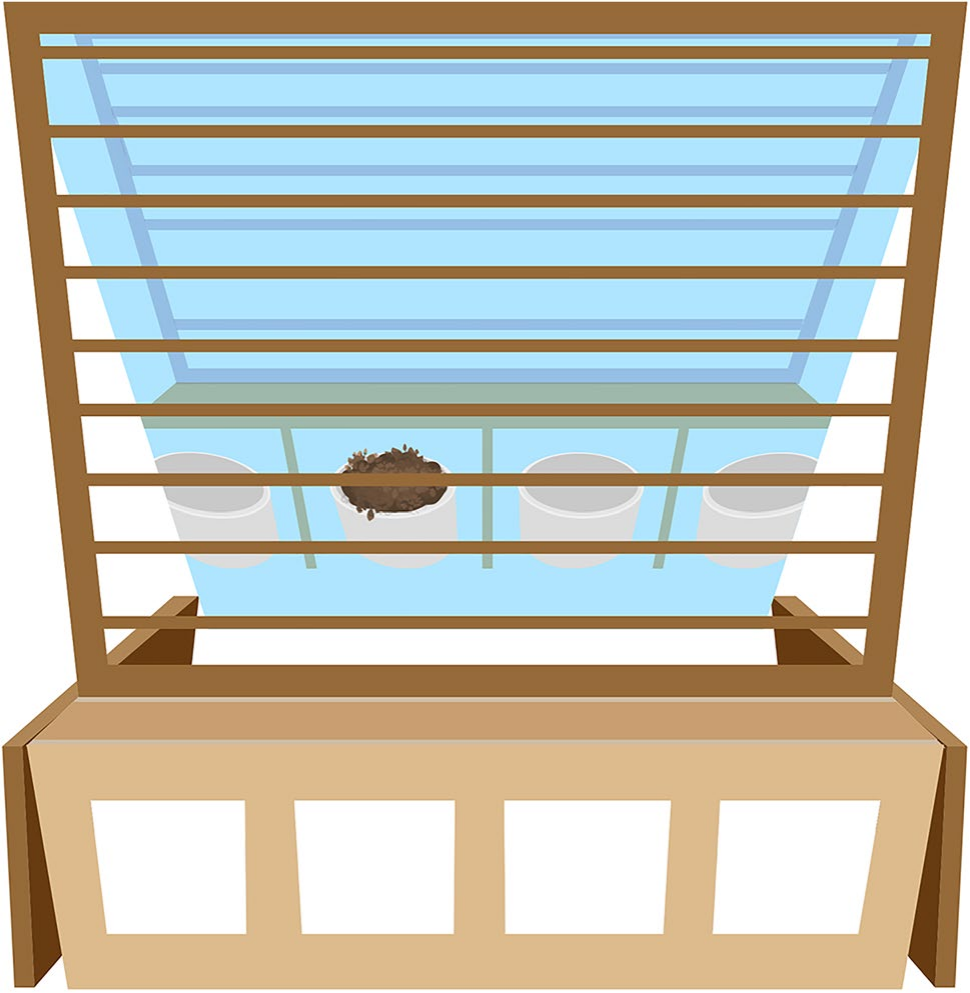
Two two-box-elements building the four-box choice apparatus. Behind the apparatus a mirror was placed vertically with its reflecting surface to the front. The sides were shielded with wooden boards and the top with grids to ensure the chickens can retrieve the food reward only through the hatches.

We used a modified version of the apparatus which Medina et al.^8^ used in their study with crows, that matched the natural pecking and searching behaviour of chickens (see Fig. 2). The apparatus consisted of two two-box-elements (two-box element: for LD: 32 × 9 × 16 cm, for JB: 25.5 × 9 × 10 cm; L × D × H), built from white coated chipboard. Each two-box-element had two separate compartments that were open to the backside. On the front side, the compartments were closed with hatches (LD: 10 × 10 cm; JB: 7.5 × 6 cm) made from cardboard.

**Figure 3:**
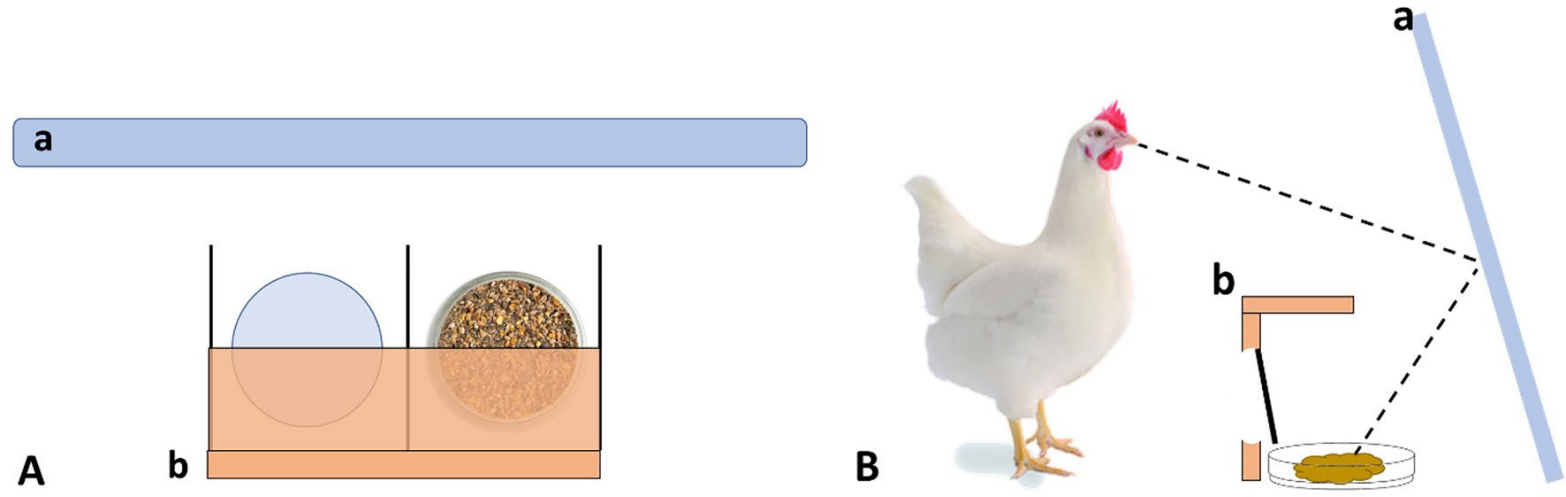
Two-box choice apparatus. (**A**) Top-view with the two separated compartments and occluding board (b) in front of the mirror (a). (**B**) Schematic cross-section of the apparatus with a baited compartment.

An occluding board (LD: 32 × 4 cm; JB: 25.5 × 4 cm) on the top of each two-box-element hid the content of each compartment from views from above (see Fig. 3A,B). A petri dish (10 cm diameter) was placed inside each compartment which could be filled with food pellets as a food reward. To correct for an olfactory bias a few food pellets were glued to the back side of each hatch. The apparatus was placed in front of a vertical mirror in 10 cm distance with the open backside of the compartments facing the mirror’s reflecting surface (see Fig. 3B). The mirror was placed vertically inside the arena, with an angle of about 75°. Wooden boards on both sides of the apparatus, as well as a wooden grid placed on top of the apparatus prevented the hens from reaching the food reward in the petri dishes in any other way than by using the hatches.

For habituation and training sessions a two-box apparatus was used. For the final test sessions two two-box-elements were placed next to each other to build a four-box choice apparatus (see Fig. 2). Note that the two-/four-box choice apparatus differed in size between breeds according to their different body sizes (mean LD: 1.543 kg, JB: 0.643 kg). For the first seven test sessions (and habituation and training as well), we used for JB hens the same apparatus as for LD hens, but it turned out to be inappropriate in size for the smaller JB hens (by observation: animals had to stretch their neck in order to see the food reward’s reflection in the mirror), although we already tried to reduce differences in body height for JB hens by placing a flat white coated chipboard on the floor (70 × 30 × 1 cm, L × D × H). From the 8th test session onwards, we used for JB hens the smaller version of the previous four-box choice apparatus.

### Procedure

The experimental procedure was subdivided into several sessions (see Table 2). Prior to each session, the hens were gently caught in their home pen and transported to the experimental room. Due to the very regular handling of the chickens, they were used to the procedure which resulted in a less than 2 min. catching-episode per animal. Hens were chosen pseudo-randomly out of the group. Chickens were carried in an upright and close to body position, according to Herborn et al.^60^, to reduce stress. A maximum of four hens was transported at the same time in different compartments (40 × 80 × 50 cm, L × D × H) in a wooden transport box with a transparent front to the experimental room. Thus, maximum duration of time an animal spend in the box did not exceed 1 h. With a testing schedule, we ensured that time spend in the box was balanced across all individuals.

**Table 2:** Overview of the sessions’ sequence, procedure and criteria for successful graduation.

Session	Phase	Procedure	Criteria
1	Habituation	10 food rewards placed inside arena	Eating within 10 min
2	Habituation	Food reward placed 10 times in front of the 2-box apparatus (hatch open)	Eating within 10 min
3	Habituation	Food reward placed 10 times inside random compartment (hatch open)	Putting head through hole to reach food, finding baited compartment, (eating within 10 min)
4	Habituation	Food reward placed 10 times inside one compartment (hatch closed)	Putting head through hatch to reach food, finding baited compartment, (eating within 10 min)
5	Habituation	10 food rewards placed in front of the mirror	Eating within 10 min
6	Habituation	Mirror + 2-box apparatus, 10 times food reward placed in random compartment (hatch closed)	Putting head through hatch to reach food, finding baited compartment (eating within 10 min.)
7	Training 1	Mirror + 2-box apparatus, 10 times food reward placed in random compartment	Finding baited compartment, eating 10 food rewards
8	Training 2	Mirror + 2-box apparatus, 10 times food reward placed in random compartment	Finding baited compartment, eating 10 food rewards
9	Training 3	Mirror + 2-box apparatus, 10 times food reward placed in random compartment	Finding baited compartment, eating 10 food rewards
10	Control with non-reflective surface	Non-reflective surface, 2 × 5 min., first right then left compartment baited	Extracting reward within 2 min
11+	Test sessions	Mirror + 4-box apparatus, 10 trials, food reward placed in compartment pseudo-randomly out of the bird’s view	Finding baited compartment, eating within 10 min

Each hen was tested in one session per day and had to meet a predefined criterion to solve the session successfully. If the criterion could not be met within a session, this session was repeated on the following day, with a maximum number of three repetitions. Each session consisted of ten trials and ended either when a hen solved the ten trials successfully (retrieved ten food rewards) or when 10 min exceeded.

#### Habituation

Habituation consisted of six consecutive sessions to habituate the animals to the experimental set-up and procedure. During habituation, hen and experimenter stayed simultaneously inside the arena to make the hen becoming familiar with the presence of the experimenter in close distance.

For the 1st habituation session, ten food rewards were placed consecutively in petri dishes inside the arena and had to be retrieved within 10 min to pass the session. During the following habituation sessions 2–4, hens were trained to use the two-box choice apparatus. In the 2nd habituation session, the two-box choice apparatus was introduced, and a food reward was placed ten consecutive times in a random order in one of two petri dishes in front of the apparatus (hatches were fixed in an open position). Hens had to eat all food rewards within 10 min to pass this session and continue with the next on the following day. In the 3rd session, the two petri dishes were placed inside the two compartments of the two-box apparatus with the hatches still open and a food reward was placed ten consecutive times randomly in one of the petri dishes. This required the hens to insert their head into one compartment to retrieve the food. The procedure of the 4th habituation session was the same as in session 3, but with the hatches being closed. For training the hens to peck against the hatch to open it (and thus retrieve the food reward), the experimenter tipped multiple times against the hatches with his finger until the hen used its beak to peck against the hatch and thus open it. In the 5th session, the apparatus was replaced by the mirror, which was fixed in a vertical position with an angle of 75° (see Fig. 3B). Ten food rewards in petri dishes were placed consecutively in front of the mirror’s reflecting surface and had to be eaten within ten minutes to pass the session. For the 6th and last session of habituation the two-box choice apparatus was placed with a distance of 10 cm in front of the mirror’s reflecting surface. The petri dishes were placed inside the two compartments, hatches remained in a closed position and restrictions in form of wooden grids on both sides and the top of the apparatus prevented the chickens from reaching the food reward from another side than through the hatches. In ten consecutive trials a food reward was placed randomly in one of the two petri dishes.

During each habituation session, the chicken was permanently present inside the arena and was able to observe the experimenter baiting the compartments, in contrast to the test session where the hen waited outside of the arena.

#### Training

Habituation was followed by three training sessions^8^, which were conducted on three consecutive days with each hen. In each of the three training sessions, a food reward was placed ten consecutive times randomly inside one of the two compartments of the two-box apparatus in front of the mirror. Each hen stayed inside the arena for the whole training session and thus, could observe which compartment has been baited. The training sessions should ensure that all hens are equally skilled in extracting the food reward from the two-box apparatus. Each session ended after ten food rewards have been extracted or 10 min elapsed.

#### Control with non-reflective surface

Following Medina et al.^8^, a control session with a non-reflective surface was conducted after the last training session and prior to final sessions with each hen. The control session aimed to control for the hen’s motivation to participate and extract a food reward. Set-up was the same as for training sessions except for the mirror being reversed, meaning the reflective surface turned to the back. Thus, chickens could only see the opaque grey side of the mirror and not the food reward’s reflection in the mirror. The control with the non-reflective surface consisted of two 5-min-trials, separated by a pause of one minute. In the first trial the right compartment of the two-box apparatus was baited, in the second trial we baited the left compartment. In contrast to Medina et al.^8^, all hens extracted the food reward within two minutes and did not need any additional motivational stimuli. During the pause of 1 min between the two trials, the chicken was removed from the arena and sat on a chair inside the experimental room, where it could not observe the experimenter baiting the compartment.

#### Test sessions: mirror-mediated spatial location task

For the mirror-mediated spatial location task, two two-box choice apparatus elements were combined and arranged side by side to form the four-box choice apparatus (see Fig. 2). As before, each hen was tested in one session per day, subdivided into ten consecutive trials. In each trial, one of the four compartments was baited pseudo-randomly, resulting in each compartment being baited 2.5 times per session on average and none being baited in two consecutive trials. A trial ended 10 s after a hen retrieved the food reward, then the hen was removed from the arena and placed on a chair outside of the arena. When the next compartment was baited and remaining food pellets have been removed from the last baited compartment, the hen was again placed with a distance of about 50 cm in front of the four-box choice apparatus. As before, the experimenter stayed inside the arena during the sessions, but on the opposite arena site of the apparatus and out of the animal’s line of sight to not disturb it. A session ended when a hen retrieved the food reward ten consecutive times by the first or subsequent tries. The arena was cleaned after each session.

### Data analysis

In this study, the natural exploration and pecking behaviour of chickens had to be taken into consideration for defining the criterion of success. Thus, a trial was scored as successful when the baited compartment was the first compartment in which a hen inserted its head by pecking against the hatch. Data acquisition was done by analysing the video recordings manually according to the sequence in which the hens inserted their heads into the different compartments, numbers of successful and unsuccessful trials, numbers of searches in the last baited compartment, latencies (time until first step) and extraction times (time until retrieving food reward). A binomial test was carried out to determine the minimum number of successful trials out of ten trials (within one session) that differs significantly from chance. Each block of ten trials was tested for significance, resulting in a minimum number of six correct trials during a ten-trial-session to differ significantly from chance and thus to suffice to solve the whole session successfully (probability for a correct trial by chance is at 25%; 6 successful trials: 1-sided binomial test: *P* = 0.016). Sessions were terminated for a hen when the hen either reached the criterion of six or more successful trials within one session or when 12 sessions elapsed without reaching the criterion (for JB termination of sessions was after 19 sessions due to the first 7 sessions with the not-fitting apparatus).

Because hens that solved the task successfully (had six or more successful trials in a ten-trial-session) needed different numbers of sessions until they reached the criterion, we used the relative proportion of successful trials for analysis instead of the total number of successful trials.

### Statistical analysis

Graphical representation of the results was done with the program SigmaPlot 14.0 (Systat Software Inc., Chicago, IL, USA). For statistical analyses, the program SPSS® Statistics 27 (IBM Corporation, Armonk, USA) was used. Significance-level α was set at *P* ≤ 0.05. Data are presented with mean and standard deviation (M ± SD). Prior to testing all assumptions for the specific statistical testing method were checked, e.g., Shapiro Wilk test for normal distribution and Levene’s test for homogeneity of variances.

To analyse population level, we used a one-sample t-test (two-sided) and tested if the whole sample differed significantly from the level of chance (25%) regarding their mean number of successful trials and their mean number of searches in the previously baited compartment.

Analysis on the breed level was done using a univariate analysis of variance (ANOVA).

### Supplementary Information

Supplementary Information.

Supplementary Table S2.

Supplementary Table S3.

Supplementary Movie S1.

## Data Availability

All relevant data are within the paper and its Supporting Information files.
