# Supplementary material for: Domestic chickens solving mirror-mediated spatial location tasks uncovering their cognitive abilities
Source: Sci Rep. 2024 Jun 19;14:14164. doi: 10.1038/s41598-024-64743-9 (PMC11187119; doi:10.1038/s41598-024-64743-9)
Supplement: Supplementary file 1 — Supplementary Information. [file 41598_2024_64743_MOESM1_ESM.docx]

# Supplementary Material

**Table S1.** **Training.** Individual performances during the three training sessions, each session consisted of 10 trials. Summary statistics for measures of successful trials, searches in the last baited compartment, extraction times until food reward was found (M ± SE and range) and the statistical comparison (rm-ANOVA) of extraction times between the three training sessions on individual, breed and population level.

| **ID** | **Breed** | **Successful trials** | **Searches in last baited comp.** | **Extraction times 1^st^ training [s]** | | **Extraction times 2^nd^ training [s]** | | **Extraction times 3^rd^ training [s]** | | **Statistics** |
| --- | --- | --- | --- | --- | --- | --- | --- | --- | --- | --- |
|  |  |  |  | **Mean** ± **SE** | **Range** | **Mean + SE** | **Range** | **Mean + SE** | **Range** | **F**_df1, df2_, **P**, partial **η²** |
| Gerdi | LD | 66.67 % | 23.33 % | 2.50 ± 0.58 | 1 - 6 | 2.50 ± 0.56 | 1 - 6 | 3.10 ± 0.86 | 1 - 10 | F_1.157, 10.416_ = 0.393, P = 0.574,  partial η² = 0.042 |
| Gundi | LD | 63.33 % | 23.33 % | 5.50 ± 1.54 | 1 - 14 | 2.00 ± 0.39 | 1 - 5 | 2.20 ± 0.44 | 1 - 5 | F_1.039, 9.347_ = 4.761, P = 0.055,  partial η² = 0.346 |
| Helli | LD | 56.67 % | 33.33 % | 5.20 ± 1.74 | 1 - 18 | 2.00 ± 0.39 | 1 - 5 | 4.90 ± 2.49 | 1 - 27 | F_2, 18_ = 0.909, P = 0.420,  partial η² = 0.092 |
| Jenna | LD | 83.33 % | 13.33 % | 3.00 ± 1.56 | 1 - 17 | 4.70 ± 2.62 | 1 - 28 | 2.90 ± 1.36 | 1 - 15 | F_2, 18_ = 0.252, P = 0.780,  partial η² = 0.027 |
| Lissi | LD | 73.33 % | 20.00 % | 5.90 ± 2.35 | 1 - 19 | 2.30 ± 0.52 | 1 - 6 | 3.20 ± 1.66 | 1 - 18 | F_2, 18_ = 1.927, P = 0.174,  partial η² = 0.176 |
| Lucy | LD | 76.67 % | 20.00 % | 2.20 ± 0.33 | 1 - 7 | 1.50 ± 0.22 | 1 - 5 | 2.70 ± 0.36 | 1 - 11 | F_2, 18_ = 5.136, P = 0.017,  partial η² = 0.363 |
| Mila | LD | 76.67 % | 13.33 % | 1.50 ± 0.40 | 1 - 5 | 2.40 ± 0.62 | 1 - 6 | 4.20 ± 1.91 | 1 - 19 | F_1.038, 9.339_ = 1.603, P = 0.237,  partial η² = 0.151 |
| Frieda | JB | 73.33 % | 13.33 % | 2.50 ± 6.54 | 1 - 51 | 9.70 ± 5.86 | 1 - 61 | 18.10 ± 6.69 | 1 - 69 | F_2, 18_ = 0.410, P = 0.669,  partial η² = 0.044 |
| Greta | JB | 66.67 % | 16.66 % | 6.5 ± 3.28 | 1 - 31 | 16.50 ± 9.52 | 1 - 98 | 9.10 ± 3.65 | 1 - 32 | F_1.248, 11.236_ = 0.759, P = 0.482,  partial η² = 0.078 |
| Gusti | JB | 90.00 % | 6.67 % | 9.00 ± 6.20 | 1 - 64 | 23.30 ± 10.94 | 1 - 92 | 12.30 ± 6.28 | 1 - 58 | F_1.108, 9.971_ = 0.805, P = 0.403,  partial η² = 0.082 |
| Lilu | JB | 90.00 % | 13.33 % | 7.50 ± 5.00 | 1 - 52 | 2.10 ± 0.46 | 1 – 61 | 5.10 ± 2.38 | 1 - 25 | F_1.270, 11.432_ = 0.645, P = 0.475,  partial η² = 0.067 |
| Lina | JB | 66.67 % | 16.66 % | 30.90 ± 10.39 | 1 - 85 | 13.00 ± 5.94 | 2 - 61 | 7.90 ± 3.74 | 1 - 36 | F_1.236, 11.124_ = 3.1835, P = 0.066,  partial η² = 0.261 |
| Lola | JB | 76.67 % | 13.33 % | 5.30 ± 2.69 | 1 - 29 | 4.40 ± 2.10 | 1 - 23 | 4.40 ± 1.52 | 1 - 13 | F_2, 18_ = 0.067, P = 0.935,  partial η² = 0.007 |
| Lui | JB | 83.33 % | 10.00 % | 2.00 ± 0.60 | 1 - 7 | 1.50 ± 0.40 | 1 - 5 | 3.50 ± 1.27 | 1 - 11 | F_2, 18_ = 2.024, P = 0.161,  partial η² = 0.184 |
| Meg | JB | 80.00 % | 10.00 % | 7.00 ± 5.78 | 1 - 59 | 10.10 ± 5.03 | 1 - 50 | 18.80 ± 8.07 | 1 - 69 | F_2, 18_ = 0.753, P = 0.485,  partial η² = 0.077 |
| Pia | JB | 76.67 % | 13.33 % | 2.40 ± 0.54 | 1 - 6 | 20.40 ± 8.91 | 1 - 77 | 4.30 ± 2.27 | 1 - 24 | F_1.063, 9.571_ = 3.375, P = 0.096,  partial η² = 0.273 |
| Tina | JB | 80.00 % | 20.00 % | 6.90 ± 3.30 | 1 - 31 | 20.50 ± 7.32 | 1 - 57 | 10.90 ± 6.19 | 1 - 48 | F_2, 18_ = 1.214, P = 0.320,  partial η² = 0.119 |
| Trish | JB | 80.00 % | 13.33 % | 20.50 ± 9.36 | 1 - 82 | 11.80 ± 8.72 | 2 - 90 | 1.90 ± 0.38 | 1 - 4 | F_2, 18_ = 1.876, P = 0.182,  partial η² = 0.172 |
| all | LD | 70.95 % | 22.38 % | 3.69 ± 0.55 | 1 - 19 | 2.49 ± 0.41 | 1 - 28 | 3.31 ± 0.55 | 1 - 27 | F_2, 138_ = 1.566, P = 0.213,  partial η² = 0.022 |
| all | JB | 78.48 % | 13.33 % | 10.41 ± 1.84 | 1 - 85 | 12.12 ± 2.10 | 1 - 98 | 8.75 ± 1.42 | 1 - 69 | F_2, 218_ = 0.865, P = 0.423,  partial η² = 0.008 |
| all | both | 75.55 % | 17.85 % | 7.79 ± 1.17 | 1 - 85 | 8.37 ± 1.33 | 1 - 98 | 6.64 ± 0.91 | 1 - 69 | *F*_2, 358_ = 0.619, *P* = 0.539,  partial *η²* = 0.003 |

Given are the percentage of successful trials of each individual per 30 trials of training, the percentage of searches in the last baited compartment per 30 trials of training, the mean (± SE) extraction time [s] for each session of training (= 10 trials), as well as the range of extraction times [s] for each session of training.

**Movie S1. Mirror-mediated spatial location task.** Jenna was the only hen, we continued testing with even after she reached the criterion of 6/10 successful trials to see if she consistently uses the mirror – what she did remarkably, as one can see in the video attached.
